# The Prevalence of Subclinical Hypothyroidism During Early Pregnancy in Pakistan: A Cross-Sectional Study

**DOI:** 10.7759/cureus.20316

**Published:** 2021-12-10

**Authors:** Rubina Sohail, Haleema Yasmin, Nasira Tasneem, Zohra Khanum, Pushpa S Sachdeve, Sadiah A Pal, Maryam Zubair, Fauzia Fahim, Sobia Ali, Raeefuddin Ahmed

**Affiliations:** 1 Obstetrics and Gynecology, Services Institute of Medical Sciences, Lahore, PAK; 2 Obstetrics and Gynecology, Jinnah Postgraduate Medical Centre, Karachi, PAK; 3 Obstetrics and Gynecology, Pakistan Institute of Medical Sciences, Islamabad, PAK; 4 Obstetrics and Gynecology, Sir Ganga Ram Hospital, Lahore, PAK; 5 Obstetrics and Gynecology, Isra University Hospital, Hyderabad, PAK; 6 Obstetrics and Gynecology, Medicell Clinic, Karachi, PAK; 7 Obstetrics and Gynecology, Azad Jammu Kashmir Medical College, Muzaffarabad, PAK; 8 Obstetrics and Gynecology, Lady Reading Hospital, Peshawar, PAK; 9 Medical Affairs, Abbott Laboratories (Pakistan) Limited, Karachi, PAK

**Keywords:** cross-sectional, hypothyroidism, pakistan, pregnancy, sub-clinical hypothyroidism

## Abstract

Introduction

Subclinical hypothyroidism (SCH) during early pregnancy is associated with an increased risk of miscarriage or premature birth. In Pakistan, the prevalence of SCH among pregnant women is not well documented. This multi-center study aims at identifying the prevalence of SCH among Pakistani pregnant women.

Methods

A cross-sectional multi-center study was conducted over a period of 12 months. Pregnant females in the first trimester of pregnancy were recruited from the antenatal clinics of seven centers from six Pakistani cities. We assessed the frequency of SCH in pregnant females and associated risk factors.

Results

A total of 500 pregnant women were enrolled in this study. Only eight women (1.6%) had a newly-diagnosed SCH. While 1.2% of women had hyperthyroidism, 6% had known hypothyroidism, and 1% had overt hypothyroidism. Ten females (33.3%) with known hypothyroidism were receiving an inadequate dose of thyroid replacement therapy. The association between BMI and SCH was not statistically significant (p = 0.69). Moreover, we could not find any significant difference between patients with or without SCH in terms of age (p > 0.90), dyslipidemia (p = 0.157), history of miscarriage (p > 0.90), the regularity of the cycle (p > 0.90), and history of infertility (p > 0.90).

Conclusions

The frequency of undiagnosed SCH in pregnant females in the study from Pakistan was 1.6%. The rate of uncontrolled hypothyroidism was high, which raises an alarm of the potential risks of untreated thyroid disorder.

## Introduction

Thyroid hormones play a pivotal role in fetal growth and uneventful pregnancy outcomes. A plethora of physiological changes occurs in the thyroid gland to meet the increased metabolic demands during pregnancy [1]. Thyroid dysfunction is one of the most commonly reported endocrine disorders during pregnancy. The prevalence of overt hypothyroidism and pathological hyperthyroidism during pregnancy varies between 0.2% and 0.6% [2]. The current body of evidence highlights several causes and risk factors for thyroid dysfunction during pregnancy; endemic iodine deficiency and autoimmune disease are among the most common causes for overt hypothyroidism in pregnant women [3]. Thyroid dysfunction can lead to multiple adverse pregnancy outcomes, including preeclampsia and other hypertensive disorders, postpartum hemorrhage, low infant birth weight, premature birth, and fetal mortality [4]. Recently, it was reported that paternal hypothyroidism can even affect the outcomes of in vitro fertilization/intracytoplasmic sperm injection. Moreover, hypothyroidism was found to affect fetal programming leading to abnormalities in fetal neurodevelopment, behavior, and cognition [5]. Thus, early diagnosis and treatment of thyroid dysfunction during pregnancy is critical to ensure optimal maternal and fetal outcomes. Nonetheless, the thyroid disorder diagnosis may be difficult due to the pregnancy-induced laboratory changes, which simulate pathological changes in non-pregnant women [6].

The presentation of hypothyroidism during pregnancy can range from asymptomatic subclinical changes to overt myxedema. Subclinical hypothyroidism (SCH) is detected when thyroid-stimulating hormone (TSH) is elevated, with a normal circulating thyroid hormone level [7]. Although being asymptomatic, SCH can significantly impact maternal and fetal outcomes. The risk of premature delivery is significantly higher in women with SCH than in normal subjects [8]. Moreover, pregnant women with SCH had a three-folded risk of developing placental abruption and miscarriage [9]. In the United States and Europe, the prevalence of SCH among pregnant women is estimated to range from 2% to 18% [10,11]. In the relatively moderate iodine-deficient countries, like India, the reported prevalence of SCH among pregnant women was as high as 13.5%-15.1% [12]. Therefore, there is an unmet need to assess the risk of SCH in pregnant women in high-risk areas, which will help in the proper planning of a universal screening program for all pregnant women.

Pakistan is known to be one of the severely iodine-deficient countries in the region [13]. In the past few years, studies carried out by the World Health Organization (WHO) provided an alarming picture of the country's profile for iodine deficiency disorders [14]. Data regarding pregnant women with hypothyroidism in Pakistan are very limited. Since hypothyroidism can devastate both the mother and infant's well-being, it is imperative to evaluate the burden of the disease. Therefore, we aimed to estimate the frequency of SCH among pregnant women from Pakistan. The Prevalence of Subclinical Hypothyroidism During Early Pregnancy in Pakistan (PRECIOUS) study was a multi-center study that assessed the prevalence of SCH among pregnant women in six Pakistani cities.

## Materials and methods

The PRECIOUS study (NCT03036956) was a multi-center, observational, cross-sectional study that was conducted over a period of 12 months, from January 2017 to January 2018, on pregnant females attending the antenatal clinic of seven centers from six cities of Pakistan (Karachi, Hyderabad, Lahore, Islamabad, Peshawar, and Muzaffarabad). The study involved only a single visit for each patient.

Inclusion and exclusion criteria

Women were included irrespective of their gravida and parity status. Adult (18-45 years) pregnant women in the first trimester who were willing to give a blood sample for laboratory testing and provide written authorization to obtain data for the study were included. The gestational age was calculated from the last menstrual period and verified by ultrasonography. The first trimester was defined as a gestational age of no more than 13 weeks ± 7 days. Women suffering from chronic cardiac diseases, hepatic disorders, renal diseases, respiratory disorders, active systemic infection, and/or physical or mental impairment were excluded. Besides, the study excluded women with a history of non-thyroid malignancy, medication for hyperthyroidism, systemic steroid therapy, or lithium therapy.

Sample size calculation

Previous studies demonstrated that the prevalence of hypothyroidism among pregnant women from neighboring countries was 4.8% in India [15], 7.5% in China [16], and 4.6% in Iran [17]. Thus, we assumed a 5% prevalence of hypothyroidism during pregnancy. Using software Epi Info (CDC, Atlanta, GA) with a 95% confidence level and a bound-on error of 0.02, the estimated sample size was 457. This sample size was inflated by 10% to account for non-responders; a final sample size of 500 pregnant women was required.

Data collection and management

All data were entered in anonymous case report forms (CRF). Data were collected regarding maternal age, body mass index (BMI), dyslipidemia, regularity of the cycle, history of miscarriages, and history of infertility. Then, all women underwent laboratory testing for thyroid function according to the local standards of each participating center.

Study outcomes

The main parameter assessed in this study was the frequency of SCH in pregnant females. The SCH was defined as TSH > 2.8 mIU/L with normal free thyroxine (FT4) level (normal range: 0.81-1.42 ng/dL). The secondary outcomes included the prevalence of overt hypothyroidism, defined as TSH > 2.8 mIU/L and FT4 < 0.8 ng/dL, and the association between SCH and other studied parameters.

Statistical analysis

Data concerning the prevalence of thyroid disorders were presented in frequencies and percentages. Continuous variables were presented in mean ± SD, while data were represented with frequency (percentage) for qualitative variables. The Mann-Whitney nonparametric test was used to compare BMI and age distributions between women with and without SCH. Fisher's exact test was employed to compare the categorical variables between women with and without SCH. All statistical tests were two-sided, and a p-value less than 0.05 was considered statistically significant. All statistical analyses were done in SAS version 9.4 (SAS Institute Inc., Cary, NC).

## Results

A total of 500 pregnant women were enrolled in this study. The mean age of the included women was 26.9 ± 4.7 years. Nearly 29% of the women were overweight, and 13.4% were obese. A total of 95 (19%) women had a history of miscarriage, and 26 (5.2%) women had a history of irregular menstrual cycles (Table 1).

**Table 1:** Baseline characteristics of the women included in the study.

Variable		Women (N = 500)
BMI in kg/m^2^	Normal	288 (57.6)
	Overweight	143 (28.6)
	Obese	67 (13.4)
	Mean (SD)	24.69 (4.77)
Age in years	Mean (SD)	26.9 (4.7)
Dyslipidemia		9 (1.8%)
History of miscarriage		95 (19%)
Cycle	Regular	474 (94.8)
	Irregular	26 (5.2)
History of infertility		28 (5.6)

**Figure 1:**
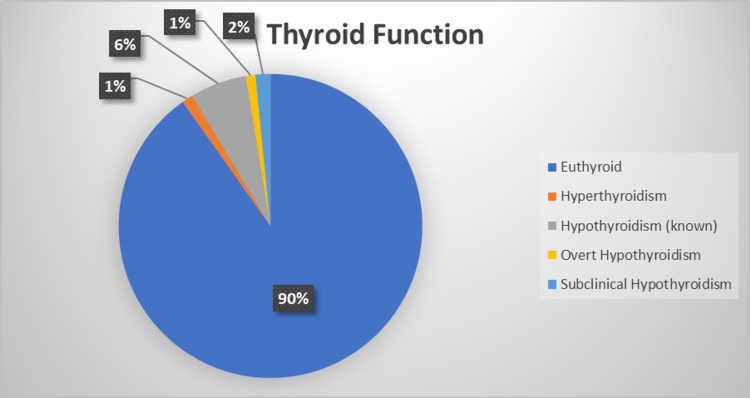
Frequency distribution of included patients based on their diagnostic outcomes.

**Figure 2:**
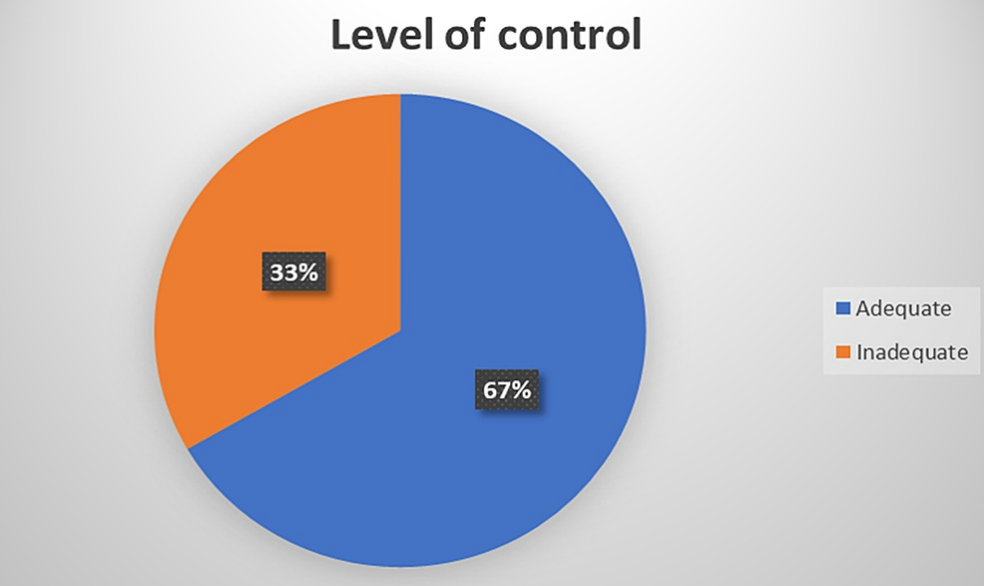
Frequency distribution of women with known hypothyroidism according to the adequacy of treatment.

Figure 1 indicates the frequency distribution of women according to their diagnostic results. The vast majority of the women (90.2%) were euthyroid, while only eight women (1.6%) had a newly-diagnosed SCH. The rest of the women were classified as known as hypothyroidism (6%), hyperthyroidism (1.2%), and overt hypothyroidism (1%). The adequacy of hypothyroidism treatment is shown in Figure 2. Ten females (33.3%) with hypothyroidism were receiving an inadequate dose of thyroid replacement therapy.

The association between BMI and SCH was not statistically significant (p = 0.69). Moreover, we could not find any significant difference between patients with or without SCH in terms of age (p > 0.90), dyslipidemia (p = 0.157), history of miscarriage (p > 0.90), the regularity of the cycle (p > 0.90), and history of infertility (p > 0.90) (Table 2).

**Table 2:** Frequency (%) and mean (SD) values of potential risk factors by subclinical hypothyroidism.

Variable		Subclinical hypothyroidism		P-value
		No (n = 492)	Yes (n = 8)	
BMI in kg/m^2^	Normal	283 (98.3)	5 (1.7)	0.690
	Overweight	140 (97.9)	3 (2.1)	
	Obese	67 (100)	0 (0.0)	
	Mean ± SD	24.7 ± 4.8	24.7 ± 4.8	
Age in years	Mean ± SD	26.9 ± 4.7	26.9 ± 5.4	>0.90
Dyslipidemia	No	415 (98.3)	7 (1.7)	0.157
	Yes	8 (88.9)	1 (11.1)	
History of miscarriage	No	398 (98.3)	7 (1.7)	>0.90
	Yes	94 (98.9)	1 (1.1)	
Cycle	Regular	466 (98.3)	8 (1.7)	>0.90
	Irregular	26 (100)	0 (0.0)	
History of infertility	No	464 (98.3)	8 (1.7)	>0.90

## Discussion

Pregnant women are more prone to hypothyroidism due to increased demands for iodine and thyroid hormones. SCH, preclinical hypothyroidism, mild hypothyroidism, and early thyroid failure are synonyms for the same condition, characterized by persistently high TSH levels, with normal circulating free thyroid hormone levels [18]. It was reported that iodine-deficient countries have an increased incidence of SCH than the iodine-sufficient countries; however, iodine's role is somewhat controversial [1]. Pakistan is known to be one of the severely iodine-deficient countries in the region. In Pakistan's general population, the prevalence of hypothyroidism and SCH was found to be 4.1% and 5.4%, respectively [19]. Nonetheless, national surveys from Pakistan regarding the prevalence of thyroid disorders among pregnant women are very limited. Besides, recent recommendations from Pakistan advocate thyroid screening for high-risk groups only, rather than a universal thyroid function screening during pregnancy [20]. Such guidelines can lead to delayed diagnosis of pregnant women with thyroid dysfunction due to symptomatic overlap and limited access to healthcare facilities in rural areas. Thus, in this study, we assessed the prevalence of SCH among pregnant women in six cities of Pakistan.

In this study, we demonstrated that the incidence of SCH and overt hypothyroidism was 1.6% and 1%, respectively. Besides, 6% of the pregnant women were found to have known hypothyroidism; out of them, 33.3% were inadequately controlled. These findings were lower than those of Talat et al., who reported that the prevalence of SCH and overt hypothyroidism in early pregnant Pakistani women was 4.44% and 4.1%, respectively [20]. A cross-sectional, multicenter study undertaken in India showed that 13.1% of pregnant women had hypothyroidism. The majority of these cases were subclinical and in the first trimester of pregnancy [12]. In a study done on pregnant women in Tehran, Iran, the prevalence of hypothyroidism was found to be 4.2%, of which 89.1% of cases were subclinical [17]. In the Democratic Republic of Congo, the prevalence of SCH was 8%, and the prevalence of isolated hypothyroxinemia was 12% [21]. A study carried out on pregnant Egyptian women targeted screening for thyroid dysfunction compared to universal screening. The results showed that targeted screening of only high-risk pregnant women resulted in missing 34.5% of women with thyroid dysfunction [22]. The prevalence of SCH among pregnant women appears to be high in European countries as well; in a study done on Caucasian women by Knight et al., the prevalence was 13.9% [23]. While the exact causes of the relatively lower prevalence of SCH in our study, compared to the previous reports, are unclear, ethnicity and environmental factors may play a role in the varying prevalence of SCH across regions. Nonetheless, our results highlighted the need for large epidemiological studies to estimate SCH in pregnant women in Pakistan. Tariq et al. highlighted the lack of awareness of Pakistani pregnant women towards hypothyroidism, which requires extensive awareness and screening programs [24].

A cumulative body of evidence highlighted several risk factors and determinants for thyroid dysfunction in pregnant women, including iodine status, ethnicity, maternal characteristics, obstetric history, and placental changes. BMI was positively correlated with serum TSH and lower FT4 levels; thus, overweight and obesity are linked with a significant increase in the risk of hypothyroidism. Besides, advanced maternal age and smoking were reported to significantly increase the risk of thyroid dysfunction during pregnancy [1]. In the present study, we observed no significant associations between the incidence of SCH and clinical characteristics of the mothers, such as BMI and maternal age. The association between SCH and obstetric history of the women was not statistically significant as well. Our findings are in line with a recent report from Saudi Arabia, which demonstrated no significant impact of maternal age or BMI on the incidence of SCH among pregnant women [25]. On the contrary, another report demonstrated a significant increase in the risk of SCH among pregnant women with high BMI [23]. Such variability in the published literature probably stems from the multifactorial nature of thyroid dysfunction in pregnant women; thus, clinical characteristics cannot be used alone to predict the risk of thyroid dysfunction.

We acknowledge that our study has some limitations. First, we could not follow the pregnancy outcomes to determine the association between them and the SCH and overt hypothyroidism. Second, our sample was selected using a convenience sampling method, which may aggravate the risk of bias.

## Conclusions

The frequency of undiagnosed SCH in pregnant females in this study from Pakistan was 1.6%. The rate of uncontrolled hypothyroidism was high, which raises an alarm of the potential risks of untreated thyroid disorder. We could not identify any risk factors of SCH. Awareness campaigns and frequent well-organized screening programs are essential to minimize the disease burden. Further research is required to evaluate the environmental risk factors that play a role in the development of SCH and result in a higher prevalence of SCH in a specific region.
